# Health Professionals' Knowledge, Attitude and Practices Regarding COVID-19 in Dessie City, Northeast Ethiopia: A Facility-Based Cross-Sectional Study

**DOI:** 10.3389/fpubh.2022.899808

**Published:** 2022-07-18

**Authors:** Tefera Alemu, Seid Legesse, Abtew Abera, Semagn Amare, Minwuyelet Maru, Birtukan Shiferaw, Assefa Missaye, Getaneh Derseh

**Affiliations:** ^1^ICAP Ethiopia, Amhara Regional Office, Bahir Dar, Ethiopia; ^2^Research and Technology Transfer Directorate, Amhara Public Health Institute Dessie Branch, Dessie, Ethiopia

**Keywords:** COVID-19, KAP, Dessie, health professionals, Ethiopia

## Abstract

**Background:**

Knowledge and attitudes are among the key drivers of social behavioral change. We assessed employed health professionals' (HPs) knowledge, attitude, and practice regarding COVID-19 in Dessie city, northeast Ethiopia.

**Methods:**

A facility-based cross-sectional study was conducted among 419 HPs working at Dessie city from 17 to 21 May 2020. The data were collected using a self-administered structured questionnaire. Knowledge, attitude, and practice are measured using 19, 16, and 8 questions, respectively. Knowledge and attitude scores are dichotomized at the 3rd quartile, while practice is using the mean value. Data entry and analysis were conducted using EpiData Manager 4.2 and SPSS 25, respectively. Three independent logistic regression analyses were carried out to determine the associated factors. We defined significant association at a *p*-value of < 0.05.

**Results:**

Out of 419 participants, 369 (88.1%) have sufficient knowledge regarding COVID-19 (95% CI: 85–91). The mean knowledge score is 16.8 with a ± 2.1 SD. Similarly, 355 (84.7%) of the HPs have a favorable attitude toward COVID-19 (95% CI: 81–87.9). The mean attitude score is 14 with ± 2.1 SD. However, practice regarding COVID-19 is adequate only in 69.7% (292) of the HPs (95% CI: 65.2–94). The mean practice score is 5.1 with a ± 1.3 SD. Sufficient knowledge is significantly associated with the type of health facility (AOR: 4.4, 95% CI: 1.4–13.3), degree and above education (AOR: 2.6, 95% Cl: 1.4–4.9), radio availability (AOR: 2.4, 95% CI: 1.3–4.7), and social media utilization (AOR: 2.3, 95% CI: 1.1–5.1). The predictors of favorable attitude are training (AOR: 3.1, 95% CI: 1.6–6.1), sufficient knowledge (AOR: 5.2, 95% Cl: 2.6–10.4), and type of health facility (AOR: 2.3, 95% CI: 1.1–5.2).

**Conclusion:**

Most HPs have sufficient knowledge and a favorable attitude regarding COVID-19. However, practice is relatively low and there remains plenty to build assertive preventive behaviors. The gap between knowledge and practice should be narrowed through an appropriate social and behavioral change communication strategy.

## Introduction

Coronavirus disease 2019 (COVID-19) is a respiratory tract infection caused by a newly discovered coronavirus, that was first recognized in Wuhan, China, in December 2019. Its mode of transmission is through respiratory droplets, direct contact, and feco-orally (1). At the moment, there is no cure for infection with the coronavirus^1^,^2^. However, the WHO has listed nine COVID-19 vaccines for emergency use to increase access to vaccination^3^.

Thus, in case of such emergencies, health professionals (HPs) are the first to encounter patients, thereby exposing themselves to a greater risk of infection. For instance, during the SARS outbreak in 2002, one-fifth of all cases were in health professionals^4^. Moreover, recently, dozens of health professionals have fallen ill with COVID-19 in Italy, China, and the United States, and many more are in quarantine after exposure to the virus, an expected but worrisome (2, 3). This is alarming, as HPs are considered frontline fighters against the disease. Also, this will fasten the spread of the disease to the community as HPs are not only at risk of acquiring infections but also of being a source of infection to patients and their families (4). On the contrary, if large numbers of HPs get infected, it will deteriorate the healthcare system's capacity to respond to a healthy contingency (2).

Many of the diseased HPs have a positive contact family indicating that they might have acquired it in the community and not in their health facility. As stated in earlier studies, HP's adherence to disease control measures can be affected by their knowledge, attitudes, and practices (KAP) toward the disease (5, 6). Therefore, the best way to reduce the risk of infection to HPs is by educating them about the COVID-19 virus, the disease it causes, how it spreads, and the way how they can use personal protective equipment (PPE) appropriately (7). Subsequently, since its first detection in China and Ethiopia, an extraordinary effort has been made by the government of Ethiopia to raise public awareness of coronavirus transmission and prevention strategies. Moreover, capacity-building training and orientations on COVID-19 have been given to the HPs working at health facilities. Yet, researchers assessing KAP toward COVID-19 among HPs across the world observed a substantial amount of deficit in the knowledge, attitude, and practice among health professionals (5, 6, 8). For instance, in one study conducted through a web-based system, 61 and 63.6% of the HPs were having poor knowledge of COVID-19 transmissions and its symptom onset, respectively (5).

Nonetheless, to the best knowledge of the investigators, there was no published evidence that assessed the knowledge, attitude, and practices of HPs regarding COVID-19 in the Ethiopian setting, particularly in the study area. Therefore, we assessed employed HP's knowledge, attitude, and practice regarding COVID-19 to identify where the gap is, which enables public health authorities to redirect their anti-corona efforts toward the gap.

## Methods

### Study Design and Period

A facility-based cross-sectional study was conducted from 17 to 21 May 2020 to assess employed health professionals' knowledge, attitude, and practice regarding COVID-19 in Dessie city.

### Study Setting and Population

The study was conducted in Dessie city administration health facilities (both government and private). It is 401 km to the northeast of Addis Ababa, the capital city of Ethiopia. Dessie had 5 sub-cities divided into 18 urban and 8 rural kebeles. According to the 2007 Central Statistical Agency report, Dessie has 285,530 population in 2021/2022, of which 49.5% are men. In 2019/2020, there were 8 health centers, 8 health posts, 2 government hospitals, 3 private hospitals, 38 private clinics, 55 private drug stores, and 4 private diagnostic laboratories. Besides, there were two governmental COVID-19 testing laboratories in the city, namely, the Amhara Public Health Institute Dessie Branch and Wollo University laboratory center (9). All health professionals working in Dessie city health facilities were the source populations. Health professionals were randomly selected and included in the study.

### Sample Size and Sampling Technique

The sample size was determined using a single population proportion formula by considering the following assumptions: 5% margin of error, 95% confidence level, and a prevalence of 50% since there was no study before. After adding a 10% nonresponse rate we obtained a minimum sample size of 424 health professionals. Using the list of health professionals as a frame, a random sampling technique was employed to select the required number of health professionals. The number of study participants in each health facility is determined based on the proportion to size allocation methods. All government health facilities are purposively included in the study, and 20 out of 38 private health facilities are selected randomly. The study is conducted in all government health centers (7), one hospital (1), all private hospitals (3), and 20 private clinics. At the time of data collection, Dessie health center and Boru Meda Specialized hospital were COVID-19 treatment centers, and HPs were not available for inclusion in the study.

### Data Collection

Data were collected through a pretested, self-administered, structured, and paper-based questionnaire, which was prepared in the Amharic language (Supplementary Material 1). The questionnaire was first prepared in English language and then translated to the Amharic version, which is the participant's mother tongue. We developed the questionnaire from different works of literature and the World Health Organization resources. The questionnaire addressed information on sociodemographic characteristics, knowledge, attitude, and practice toward COVID-19.

### Data Quality Assurance

Before the actual data collection period, pretesting of the questionnaire was conducted on 5% of the study population, and necessary modifications have been made. Training was given to data collectors/supervisors. Daily supervision was made by the investigators. At the time of data collection, respondents are briefed on the questionnaire, and the answers were given to each question raised by participants. Tool validation has been conducted, and Cronbach's alpha is found to be in the good range (α = 0.84).

### Data Analysis

Data entry and analysis were made using the EpiData Manager 4.2 and SPSS 25 software, respectively. Reverse coding of some questions was made for negatively worded questions. Logistic regression analyses have been conducted to identify factors associated with KAP regarding COVID-19. We entered a variable with a *p*-value of ≤ 0.2 in the bivariate analysis into the multivariate logistic analysis. Adjusted odds ratios with a 95% confidence interval were computed to observe the strength of association between the dependent and independent variables. A *p*-value of < 0.05 was considered statistically significant. Before the regression analysis, the model fitness was checked by the Hosmer and Lemeshow goodness-of-fit test.

### Operational Definition

#### Sufficient Knowledge

A total of 19 items are used to measure HP's knowledge regarding COVID-19. The mean knowledge score is 16.8, and those participants who scored 15 and more (3rd quartile) are classified as having sufficient knowledge. Those “Yes” responses are coded as 1 and those “No” and “I do not know” responses are taken as incorrect responses and coded as 0.

#### Favorable Attitude

A total of 16 items are used to measure participants' attitudes, and the mean attitude score is 14; those participants who scored 13 and more (3rd quartile) are classified as having favorable knowledge. Participants who answered “Agree/Yes” were considered correct responses, while those who answered “Disagree/No” and “I do not know” were taken as incorrect responses.

#### Adequate Practice

The overall practice regarding COVID-19 is measured using eight questions. The mean practice score is found to be 5.1 and scores above the mean value are taken as adequate.

### Ethics Approval and Consent to Participate

Ethical approval was obtained from Amhara Public Health Institute Ethical review committee (Protocol No: H/R/T/T/D/3/791). Permission was also obtained from concerned bodies in Dessie city. Informed oral consent is also obtained from each participant. All possible COVID-19 measures are taken to prevent cross-contamination during data collection.

## Results

### Sociodemographic Characteristics of Participants

A total of 419 participants aged >20 years were included in the study with a response rate of 98.8%. The mean age of participants was 32 years with ± 8.9 standard deviation. Half of the participants are male and about 61.3% were married. More than half (54.9%) of the participants are at least first-degree holders, while the rest 45.1% had a diploma in health science fields. The majority (88.8%) of the participants had a television in their houses, and 86.6% of them are currently social media users. Notably, 40% of the participants are working at health centers, while the rest 37.7% and 21.7% are working in hospitals and private clinics, respectively (Table 1).

**Table 1:** Sociodemographic characteristics of health professionals in Dessie city, northeast Ethiopia (*N* = 419).

			**Knowledge**		**Attitude**		**Practice**	
**Variables**	**Category**	**Frequency (%)**	**Sufficient**	**Insufficient**	**Favorable**	**Unfavorable**	**Adequate**	**Inadequate**
Age	20–30 Years	254 (60.6)	220 (86.6)	34 (13.4)	216 (85)	38 (15)	170 (66.9)	84 (33.1)
	31–40 Years	104 (24.8)	92 (88.5)	12 (11.5)	86 (82.7)	18 (17.3)	77 (74)	27 (26)
	41 and above	61 (14.6)	57 (93.4)	4 (6.6)	53 (86.9)	8 (13.1)	45 (73.8)	16 (26.2)
Sex	Male	212 (50.6)	194 (91.5)	18 (8.5)	181 (85.4)	31 (14.6)	146 (68.9)	66 (31.1)
	Female	207 (49.4)	175 (84.5)	32 (15.5)	174 (84.1)	33 (15.9)	146 (70.5)	61 (29.5)
Religion	Orthodox	246 (58.7)	212 (86.2)	34 (13.8)	214 (87)	32 (13)	174 (70.7)	72 (29.3)
	Muslim	164 (39.1)	148 (90.2)	16 (9.8)	133 (81.1)	31 (18.9)	113 (68.9)	51 (31.1)
	Protestant	9 (2.1)	9 (100)	0	8 (88.9)	1 (11.1)	5 (55.6)	4 (44.4)
Marital status	Married	257 (61.3)	227 (88.3)	30 (11.7)	218 (84.8)	39 (15.2)	183 (71.2)	74 (28.8)
	Single	145 (34.6)	130 (89.7)	15 (10.3)	121 (83.4)	24 (16.6)	96 (66.2)	49 (33.8)
	Divorced/windowed	17 (4.1)	12 (70.6)	5 (29.4)	16 (94.1)	1 (5.9)	13 (76.5)	4 (23.5)
Level of education	Diploma	189 (45.1)	156 (82.5)	33 (17.5)	153 (81)	36 (19)	134 (70.9)	55 (29.1)
	Degree & above	230 (54.9)	213 (92.6)	17 (7.4)	202 (87.8)	28 (12.2)	158 (68.7)	72 (31.3)
Experience	0–5 Years	152 (36.3)	134 (88.2)	18 (11.8)	130 (85.5)	22 (14.5)	101 (66.4)	51 (33.6)
	6–10 Years	148 (35.3)	125 (84.5)	23 (15.5)	122 (82.4)	26 (17.6)	102 (68.9)	46 (31.1)
	≥11 Years	119 (28.4)	110 (92.4)	9 (7.6)	103 (86.6)	16 (13.4)	89 (74.8)	30 (25.2)
Owner of HF	Non-governmental HFs	154 (36.8)	138 (89.6)	16 (10.4)	136 (88.3)	18 (11.7)	116 (75.3)	38 (24.7)
	Governmental HFs	265 (63.2)	231 (87.2)	34 (12.8)	219 (82.6)	46 (17.4)	176 (66.4)	89 (33.6)
Type of HF	Health Center	170 (40.6)	144 (84.7)	26 (15.3)	133 (78.2)	37 (21.8)	112 (65.9)	58 (34.1)
	Clinic	91 (21.7)	87 (95.6)	4 (4.4)	79 (86.8)	12 (13.2)	63 (69.2)	28 (30.8)
	Hospital	158 (37.7)	138 (87.3)	20 (12.7)	143 (90.5)	15 (9.5)	117 (74.1)	41 (25.9)
Television availability	No	47 (11.2)	43 (91.5)	4 (8.5)	37 (78.7)	10 (21.3)	29 (61.7)	18 (38.3)
	Yes	372 (88.8)	326 (87.6)	46 (12.4)	318 (85.5)	54 (14.5)	263 (70.7)	109 (29.3)
Radio availability	No	236 (56.3)	154 (84.2)	29 (15.8)	153 (83.6)	30 (16.4)	118 (64.5)	65 (35.5)
	Yes	183 (43.7)	215 (91.1)	21 (8.9)	202 (85.6)	34 (14.4)	174 (73.7)	62 (26.3)
Social media utilization	No	56 (13.4)	44 (78.6)	12 (21.4)	48 (85.7)	8 (14.3)	41 (73.2)	15 (26.8)
	Yes	363 (86.6)	325 (89.5)	38 (10.5)	307 (84.6)	56 (15.4)	251 (69.1)	112 (30.9)

### Knowledge Regarding COVID-19

A total of 19 items are used to measure HP's knowledge regarding COVID-19. The mean knowledge score is 16.8 with ± 2.1 standard deviations, ranging from 6 up to 19 scores. Regarding specific knowledge items, more than 90% of the health professionals know the major COVID-19 symptoms like fever (95%), dry cough (90.7%), and shortness of breathing (92.6%). Similarly, most of the respondents know the major COVID-19 transmission routes like respiratory droplets (94.7%), direct (97.1%), and indirect contacts (92.4%). Furthermore, most HPs know all the COVID-19 prevention methods like frequent hand washing with soap (98.8%), physical distancing (97.1%), staying at home (95.9%), sanitizer utilization (95.7%), and facemask utilization (90.5%). Besides, 84% of the participants mentioned that the elderly and people with chronic illness are at higher risk of severe illness and bad outcomes. Also, 91.4% of the HPs mentioned that asymptomatic carriers can transfer the virus and 97.6% of them believe that more than one prevention method is important for COVID-19 prevention. In general, 88.1% (369) of health professionals have sufficient knowledge regarding COVID-19 (95% CI: 85–91) (Table 2).

**Table 2:** Health professionals' knowledge regarding COVID-19 in Dessie city, northeast Ethiopia (*N* = 419).

**Knowledge Items**	**Category**	**Frequency**	**Percent**
Received COVID-19 training/orientation	Yes	174	41.5
	No	245	58.5
COVID-19 distribution	Pandemic	337	80.4
	Epidemic	81	19.6
Know toll-free line	Yes	298	71.1
	No	120	28.6
**COVID-19 Symptoms**			
Fever	Yes	398	95
	No	21	5
Dry cough	Yes	380	90.7
	No	39	9.3
Shortness of breathing	Yes	388	92.6
	No	31	7.4
Common cold-like symptoms	Yes	331	79
	No	88	21
Fatigue and myalgia	Yes	291	69.5
	No	128	30.5
Loss of appetite	Yes	250	59.7
	No	169	40.3
**Mode of transmission**			
Respiratory droplets	Yes	397	94.7
	No	22	5.3
Direct contact	Yes	407	97.1
	No	12	2.9
Indirect contact	Yes	387	92.4
	No	31	7.6
Eating vegetables and uncooked meats	Yes	358	85.4
	No	61	14.6
Air born	Yes	332	79.2
	No	87	20.7
**COVID-19 prevention methods**			
Handwashing with soap	Yes	414	98.8
	No	5	1.2
Physical distancing	Yes	407	97.1
	No	12	2.9
Staying home	Yes	402	95.9
	No	17	4.1
Facemask utilization	Yes	379	90.5
	No	40	9.5
Sanitizer utilization	Yes	401	95.7
	No	18	4.3
Avoiding crowded places	Yes	394	94
	No	25	6
Good respiratory hygiene	Yes	388	92.6
	No	31	7.4
Isolation and treatment	Yes	386	92.1
	No	33	7.9
Avoid touching openings	Yes	392	93.6
	No	27	6.4
**The population at risk of getting infected**			
All ages and both sex	Yes	402	95.9
	No	17	4.1
**Vulnerable to becoming severely ill or die**			
Older age group	Yes	353	84.2
	No	66	15.8
People with chronic illness	Yes	349	83.3
	No	70	16.7
**Suspected case definition**			
Symptoms plus travel history	Yes	382	91.2
	No	37	8.8
Symptoms plus contact history	Yes	389	92.8
	No	30	7.2
Symptoms plus occupational risk	Yes	334	79.7
	No	85	20.3
Symptoms only	Yes	238	56.8
	No	181	43.2
**Activities following a single case detection**			
Isolation and treatments	Yes	409	97.6
	No	10	2.4
Contact tracing and quarantine	Yes	395	94.3
	No	24	5.7
Laboratory test for contacts	Yes	390	93.1
	No	29	6.9
Disinfection of houses	Yes	366	87.4
	No	53	12.6
**Other knowledge variables**			
Are asymptomatic cases cannot transmit the virus?	No	383	91.4
	I do not know	4	1
	Yes	32	7.6
Is a single prevention method adequate?	No	409	97.6
	Yes	10	2.4
Children and young adults shouldn't take preventive measures	No	354	84.5
	I do not know	12	2.9
	Yes	53	12.6
Know the duration of quarantines	Yes	393	93.8
	No	26	6.2
Currently, a drug to cure COVID-19 is available.	No	377	90
	I do not know	36	8.6
	Yes	6	1.4
Currently, COVID-19 vaccination is available	No	372	88.8
	I do not know	43	10.3
	Yes	4	1
COVID-19 case fatality rate is 100%	No	389	92.8
	I do not know	22	5.3
	Yes	8	1.9
Supportive treatment can help patients to recover	Yes	377	90
	No	24	5.7
	I do not know	18	4.3
Is their COVID-19 death in Ethiopia?	Yes	410	97.9
	No	5	1.2
	I do not know	4	1
Knowledge classification	Sufficient	369	88.1
	Insufficient	50	11.9

### Attitude Toward COVID-19

A total of 16 items are used to measure participants' attitudes toward COVID-19. The mean attitude score is 14 with ± 2.1 standard deviations, ranging from 1 up to 16 scores. Approximately 3.6% of the HPs agree that COVID-19 will not infect/kill Ethiopian/African origins; whereas the overwhelming majority (96.9%) disagree with the idea that COVID-19 only infects elders. Likewise, 96.7% of the HPs disagree with the idea that COVID-19 will not kill children and youths, and 89.5% of them believe that COVID-19 precaution measures are important to children. Besides, eight percent of the HPs believe that only N95 face mask utilization is adequate to prevent COVID-19 infection (Table 3).

**Table 3:** Health professionals' attitude toward COVID-19 in Dessie city, northeast Ethiopia (*N* = 419).

**Attitude Items**	**Category**	**Frequency**	**Percent**
COVID-19 will not infect/kill Ethiopian/African origins	Disagree	404	96.4
	Agree	15	3.6
Do you agree that COVID-19 only infects elders?	Disagree	406	96.9
	Agree	13	3.1
Do you believe that COVID-19 will not kill children and youths?	Disagree	405	96.7
	Agree	14	3.3
No need to bother about COVID-19 precaution measures, leave it for God/Allah	Disagree	375	89.5
	Agree	44	10.5
Do you think that only N95 face mask utilization is adequate to prevent COVID-19 infection?	Disagree	385	91.9
	Agree	34	8.1
Do you think those cultural medications can prevent and/or cure COVID-19?	Disagree	364	86.9
	Agree	24	5.7
	I don't know	31	7.4
Do you think that avoiding mass gatherings will prevent coronavirus infection?	Agree	342	81.6
	Disagree	77	18.4
Do you believe that frequent hand washing and using sanitizers can prevent coronavirus infection?	Agree	369	88.1
	Disagree	50	11.9
Do you believe that staying at home can prevent coronavirus infection?	Agree	373	89
	Disagree	46	11
Do you believe that maintaining social distancing can prevent coronavirus infection?	Agree	389	92.8
	Disagree	30	7.2
Do you advise coronavirus prevention strategies for your friends and families?	Yes	412	98.3
	No	7	1.7
Do you report a suspected case of your intimate friend to a health authority for quarantine?	Yes	409	97.6
	No	10	2.4
Are you committed to supporting the government in fighting against COVID-19?	Yes	401	95.7
	No	18	4.3
Are you ready to procure COVID-19 prevention commodities even at a high cost?	Yes	296	70.6
	No	72	17.2
	I do not know	51	12.2
Do you agree that COVID-19 is a preventable disease?	Agree	306	73
	Disagree	82	19.6
	I don't know	31	7.4
Do you agree that Ethiopia can win the battle against the COVID-19 virus?	Agree	250	59.7
	Disagree	80	19.1
	I don't know	89	21.2
Attitude classification	Favorable	355	84.7
	Unfavorable	64	15.3

Most of the HPs agree that avoiding mass gatherings (81.6%), frequent hand washing (88.1%), staying at home (89%), and maintaining a social distance (92.8%) will prevent COVID-19 infection. About 86.9% of the HPs disagree with the idea of cultural medications to prevent and/or cure COVID-19 infection (Table 3). Nearly all (98.3%) health professionals agree to advise their friends and families about COVID-19-related issues. Besides, 97.6% of them are committed to reporting a suspected COVID-19 case to a health authority for quarantine. Also, 70.6% of the HPs are ready to procure COVID-19 prevention commodities and supplies even at a high cost (Table 3). Overall, 355 (84.7%) of the HPs have a favorable attitude toward COVID-19 (95% CI: 81–87.9).

### Practice Regarding COVID-19

This study revealed that health professionals are uniformly implementing the major COVID-19 preventive measure in their day-to-day life. As reported by HPs, 94% of them are frequently washing their hands with soap, but only 87.8% utilize soap at each handwashing event. Similarly, even though 94.7% of the HPs reported sanitizer utilization, only 83.3% have sanitizer at data collection time. Despite the report to use facemasks by 86.4% of the participants, we found a regular utilization of facemasks in 51.8% of the HPs. Also, 95.5% of the HPs mentioned that they avoided handshaking, 85.7% are keeping their physical distance, and 61.3% have avoided visiting crowded places. Unprecedently, almost 5% of the study participants reported that they are not implementing any of the recommended COVID-19 precautionary methods. In general, the overall practice regarding COVID-19 is measured using eight questions, and the mean practice score is 5.1 with ± 1.3 standard deviation, which ranges from 2 up to 8 scores. Overall, 69.7% (292) of health professionals have adequate practice regarding COVID-19 (95% CI: 65.2–94) (Table 4).

**Table 4:** Health professionals' practice on COVID-19 preventive measures in Dessie city, northeast Ethiopia (*N* = 419).

**Practice Items**	**Category**	**Frequency**	**Percent**
**Which preventive measure are you mostly practicing?**			
Frequent hand washing	Yes	394	94
	No	25	6
Alcohol-based hand rub	Yes	397	94.7
	No	22	5.3
Facemask	Yes	362	86.4
	No	57	13.6
Avoided handshaking	Yes	400	95.5
	No	19	4.5
Keeping physical distance	Yes	359	85.7
	No	60	14.3
Staying at home	Yes	187	44.6
	No	232	55.4
Good respiratory hygiene's	Yes	372	88.8
	No	47	11.2
Avoided touching eyes, nose and mouth	Yes	380	90.7
	No	39	9.3
Avoided taking public transportations	Yes	199	47.5
	No	220	52.5
I am not practicing any method	Yes	20	4.8
	No	399	95.2
In recent days, have you visited any crowded places?	No	257	61.3
	Yes	162	38.7
Are you keeping your physical distancing?	Always	115	27.4
	Sometimes	258	61.6
	No	46	11
Are you using a facemask outside the home?	Always	217	51.8
	Sometimes	165	39.4
	No	37	8.8
Hand washing increment as compared to the pre-corona area	The same	17	4.1
	Two times	43	10.3
	Three times	86	20.5
	Four times	84	20
	Five times	125	29.8
	Six and above	64	15.3
Frequency of soap utilization	Always	368	87.8
	Sometimes	44	10.5
	Never	7	1.7
Do you have sanitizer now?	Yes	349	83.3
	No	70	16.7
Is their patient triage in your facility?	Always	187	44.6
	Sometimes	123	29.4
	No	109	26

### Factors Associated With COVID-19 Knowledge and Attitude

In simple binary logistic regression, we recruited (*p*-value < 0.2) the variables age, sex, marital status, type of health facility, level of education, presence of radio, and social media utilization for the final model. In multivariable logistic regression analysis, knowledge regarding COVID-19 is significantly associated with the level of education, type of health facility, the presence of radio, and social media utilization.

The type of health facility is significantly associated with sufficient knowledge regarding COVID-19. Hence, health professionals working in private clinics are four times more likely to have sufficient knowledge than those working in government health centers (AOR: 4.4, 95% CI: 1.4–13.3). Health professionals who have a first degree and above education are 2.6 times more likely to have sufficient knowledge as compared to diploma holders (AOR: 2.6, 95% Cl: 1.4–4.9). The odd of sufficient knowledge is also increased over 2-fold among respondents having a radio in their house (AOR: 2.4, 95% CI: 1.3–4.7). In addition, health professionals who are utilizing social media are 2.3 times more likely to have sufficient knowledge as compared to their counterparts (AOR: 2.3, 95% CI: 1.1–5.1; Table 5).

**Table 5:** Factors associated with sufficient knowledge and favorable attitude regarding COVID-19 in Dessie city, northeast Ethiopia (*N* = 419).

**Variables**	**Category**	**Knowledge**				**Attitude**			
		**Sufficient knowledge**	**Insufficient knowledge**	**COR (95%CI)**	**AOR (95%CI)**	**Favorable attitude**	**Unfavorable attitude**	**COR (95%CI)**	**AOR (95%CI)**
Age	20–30 Years	220 (86.6)	34 (13.4)	1	1	216 (85)	38 (15)	1	
	31–40 Years	92 (88.5)	12 (11.5)	1.2 (0.6–2.4)	1.4 (0.6–3.2)	86 (82.7)	18 (17.3)	0.8 (0.5–1.6)	
	41 and above	57 (93.4)	4 (6.6)	2.2 (0.8–6.5)	3.7 (1.1–12.9)	53 (86.9)	8 (13.1)	1.2 (0.5–2.6)	
Sex	Male	194 (91.5)	18 (8.5)	1	1	181 (85.4)	31 (14.6)	1	
	Female	175 (84.5)	32 (15.5)	0.5 (0.3–0.9)	0.8 (0.4–1.6)	174 (84.1)	33 (15.9)	0.9 (0.5–1.5)	
Religion	Orthodox	212 (86.2)	34 (13.8)	1		214 (87)	32 (13)	1	1
	Muslim	148 (90.2)	16 (9.8)	1.5 (0.8–2.8)		133 (81.1)	31 (18.9)	0.6 (0.4–1.1)	0.6 (0.3–1.1)
	Protestant	9 (100)	0			8 (88.9)	1 (11.1)	1.2 (0.1–9.9)	1.1 (0.1–10)
Marital status	Married	227 (88.3)	30 (11.7)	1	1	218 (84.8)	39 (15.2)	1	
	Single	130 (89.7)	15 (10.3)	1.2 (0.6–2.2)	1.4 (0.7–3.0)	121 (83.4)	24 (16.6)	0.9 (0.5–1.6)	
	Divorced/windowed	12 (70.6)	5 (29.4)	0.3 (0.1–1.0)	0.4 (0.1–1.6)	16 (94.1)	1 (5.9)	2.9 (0.4–22.2)	
Level of education	Diploma	156 (82.5)	33 (17.5)	1	1	153 (81)	36 (19)	1	1
	Degree and above	213 (92.6)	17 (7.4)	2.7 (1.4–4.9)	2.6 (1.4–4.9)**	202 (87.8)	28 (12.2)	1.7 (1.0–2.9)	1.3 (0.7–2.4)
Experience	0–5 Years	134 (88.2)	18 (11.8)	1		130 (85.5)	22 (14.5)	1	
	6–10 Years	125 (84.5)	23 (15.5)	0.7 (0.4–1.4)		122 (82.4)	26 (17.6)	0.8 (0.4–1.5)	
	≥11 Years	110 (92.4)	9 (7.6)	1.6 (0.7–3.8)		103 (86.6)	16 (13.4)	1.1 (0.5–2.2)	
Owner of HF	Non-governmental HFs	138 (89.6)	16 (10.4)	1		136 (88.3)	18 (11.7)	1	
	Governmental HFs	231 (87.2)	34 (12.8)	0.8 (0.4–1.5)		219 (82.6)	46 (17.4)	1.6 (0.9–2.9)	
Type of HF	Health Center	144 (84.7)	26 (15.3)	1	1	133 (78.2)	37 (21.8)	1	1
	Clinic	87 (95.6)	4 (4.4)	3.9 (1.3–11.6)	4.4 (1.4–13.3)**	79 (86.8)	12 (13.2)	1.8 (0.9–3.7)	2.3 (1.1–5.2)*
	Hospital	138 (87.3)	20 (12.7)	1.2 (0.7–2.3)	1.1 (0.6–2.1)	143 (90.5)	15 (9.5)	2.7 (1.4–5.1)	3.6 (1.8–7.2)**
Television availability	No	43 (91.5)	4 (8.5)	1		37 (78.7)	10 (21.3)	1	
	Yes	326 (87.6)	46 (12.4)	0.7 (0.2–1.9)		318 (85.5)	54 (14.5)	1.6 (0.8–3.4)	
Radio availability	No	154 (84.2)	29 (15.8)	1	1	153 (83.6)	30 (16.4)	1	
	Yes	215 (91.1)	21 (8.9)	1.9 (1.1–3.5)	2.4 (1.3–4.7)**	202 (85.6)	34 (14.4)	1.2 (0.7–2.0)	
Social media utilization	No	44 (78.6)	12 (21.4)	1	1	48 (85.7)	8 (14.3)	1	
	Yes	325 (89.5)	38 (10.5)	2.3 (1.1–4.8)	2.3 (1.1–5.1)*	307 (84.6)	56 (15.4)	0.9 (0.4–2.0)	
Training/orientation	No	214 (87.3)	31 (12.7)	1		198 (80.8)	47 (19.4)	1	1
	Yes	155 (89.1)	19 (10.9)	1.2 (0.6–2.2)		157 (90.2)	17 (9.8)	2.2 (1.2–4.0)	3.1 (1.6–6.1)**
Knowledge	Insufficient					29 (58)	21 (42)	1	1
	Sufficient					326 (88.3)	43 (11.7)	5.5 (2.9–10.5)	5.2 (2.6–10.4)**

**P-value < 0.01.

*P-value < 0.05.

In the second binary logistic regression, knowledge, religion, type of health facility, level of education, and training on COVID-19 are selected for the final model. However, in multivariable logistic regression analysis, the only variables independently associated with attitude are knowledge, type of working facility, and training on COVID-19.

As a result, the odd of a favorable attitude regarding COVID-19 is 3.1 times higher among health professionals who took COVID-19 training/orientation than their counterparts (AOR:3.1, 95% CI: 1.6–6.1). The presence of sufficient knowledge was another factor significantly associated with a favorable attitude regarding COVID-19. Consequently, health professionals who have sufficient knowledge about COVID-19 are five times more likely to have a favorable attitude regarding COVID-19 (AOR: 5.2, 95% Cl: 2.6–10.4). Besides, health professionals who are employed in private clinics are 2.3 times more likely to have a favorable attitude regarding COVID-19 than health professionals employed in government health centers (AOR: 2.3, 95% CI: 1.1–5.2). This also extends to health professionals working at hospitals that have higher odds of favorable attitudes than their counterparts working at health centers (AOR: 3.6, 95% CI: 1.8–7.2; Table 5).

## Discussion

Our study revealed a high level of knowledge and a favorable attitude in the study area. However, in our study, practice is found to be inadequate in 30% of the participants, which shows that good knowledge and a favorable attitude do not necessarily lead to social behavioral change. A similar gap between knowledge and practice is also observed in a study conducted in the northeast part of Ethiopia (10, 11). Further study and evaluation are warranted to investigate the reason behind low infection prevention practice despite the high level of knowledge and favorable attitude in the area. However, the applications of an appropriate social and behavioral change communication strategy with an increased access to personal protective equipment and mandatory regulations can narrow the gap between knowledge and practice. Still, a high number of health professionals are practicing the major COVID-19 precautions like handwashing, sanitizer utilization, face mask, and avoiding handshaking.

Surprisingly, this study revealed that approximately 5% of the participants are not practicing any of the prevention methods. Also, nearly 4% of the HPs believe that COVID-19 will not infect/kill Ethiopian/African origins. Besides, 10.5% of them believe that people should not bother about COVID-19 precaution measures. That is why around 20–30% of the HPs are currently hesitant/delayed to take the COVID-19 vaccine once it became available (12, 13). The reason might be low perceived susceptibility to getting infected and/or severe COVID-19 outcomes, or it might show desperation to combat the virus, but it is largely undiscovered and similar findings are rare in literature. Overall, 15% of the HPs have an unfavorable attitude regarding COVID-19, which is alarming from the pandemic point of view as well as the overcrowded healthcare setups and living conditions in Dessie city. Such negative attitudes by HPs will further accelerate the spread of disease in the community, especially the omicron variant of the virus. In this regard, health facility governing bodies should seriously examine and take responsibility to execute COVID-19 preventive methods in the entire healthcare system. At present, special emphasis should be given to COVID-19 vaccination uptakes by all HPs, as the course of the disease has drastically changed with vaccines and failure to do so will lead to devastating effects on public health and hinder the healthcare system's ability to accommodate the challenges of the pandemic (14). Nonetheless, the 85% favorable attitude in our study is higher than the 70.7% pooled estimate report in Ethiopia (15) and the 64% report from the northeast Ethiopia (10).

We also identified the predictors of knowledge and attitudes regarding COVID-19. The first variable is degree and above level of education that influences COVID-19 knowledge with more than 2-fold. A similar finding was also noted in southern Ethiopia (16). The other variable is working in private clinics, which increases the odds of sufficient knowledge by more than 4-fold as compared to participants working in government health centers. There might be more frequent staff orientations and emphasis on preventive measures at private clinics than at government health facilities, which can contribute to the current knowledge difference. Besides, social media utilization like Facebook is found to be a significant factor that influences participants' knowledge regarding COVID-19. The same finding is also reported from a multicentered study conducted in Ethiopia (17). This implies that the internet and social media can play a pivotal role in timely public health risk communication like COVID-19, which hinders mass classroom discussions and meetings. During similar emergencies, virtual means of communication like training through zoom meetings should be adopted, even for health workers' continuous professional growth in development situations. In this study, participating in COVID-19-related training is found to be a predictor of favorable attitude, which is like other studies conducted in southern Ethiopia (16) and Venezuelan HPs (18). Similarly, working in hospitals and clinics becomes significantly associated with a favorable attitude than working in health centers. This might be related to the easy accessibility of COVID-19 information through bedsides, patient rounds, case presentations, and discussions in hospitals and private clinics than in government health centers. As a limitation, our findings might be overreported by participants as it is a self-administered way of data collection.

## Conclusion

Most health professionals have a high level of knowledge and a favorable attitude regarding COVID-19. However, practice is relatively low and there remains plenty to build assertive preventive behaviors. Factors associated with having sufficient knowledge are the type of health facility, degree and above educational status, social media utilization, and radio availability, while favorable attitude is significantly associated with COVID-19-related training, type of health facility, and having sufficient knowledge. The gap between knowledge and practice among HPs can be narrowed through the application of an appropriate social and behavioral change communication strategy and mandatory regulations.

## Data Availability Statement

Original datasets are available in a publicly accessible repository: This data can be found here: https://datadryad.org/stash/share/g9_e95UE7T-YHHA50pxSBkBnHyB7Y3h-ZHcm9Smv03I.

## Author Contributions

All authors listed have made a substantial, direct, and intellectual contribution to the work and approved it for publication.

## Funding

The APHI Dessie Branch has covered data enumeration expenses.

## Conflict of Interest

The authors declare that the research was conducted in the absence of any commercial or financial relationships that could be construed as a potential conflict of interest.

## Publisher's Note

All claims expressed in this article are solely those of the authors and do not necessarily represent those of their affiliated organizations, or those of the publisher, the editors and the reviewers. Any product that may be evaluated in this article, or claim that may be made by its manufacturer, is not guaranteed or endorsed by the publisher.

## Footnotes

^1^ https://www.britannica.com/story/is-there-a-cure-for-coronavirus

^2^ https://www.who.int/docs/default-source/coronaviruse/key-messages-and-actions-for-covid-19-prevention-and-control-in-schools-march-2020.pdf?sfvrsn=baf81d52_4

^3^ https://www.who.int/news/item/17-12-2021-who-lists-9th-covid-19-vaccine-for-emergency-use-with-aim-to-increase-access-to-vaccination-in-lower-income-countries

^4^ https://www.theverge.com/2020/3/5/21166088/coronavirus-covid-19-protection-doctors-nurses-health-workers-risk
