# Supplementary material for: Health Professionals' Knowledge, Attitude and Practices Regarding COVID-19 in Dessie City, Northeast Ethiopia: A Facility-Based Cross-Sectional Study
Source: Front Public Health. 2022 Jul 18;10:899808. doi: 10.3389/fpubh.2022.899808 (PMC9341290; doi:10.3389/fpubh.2022.899808)
Supplement: Supplementary file 1 [file Data_Sheet_1.pdf]

Questionnaire for Health Professionals (KAP on COVID-19)

Part I. Socio demographic characteristic

1. Name of health facility………………………………Date …..…/09/202

2. Age_________

3. Sex: A, Male B, Female

4. Religion A. Orthodox B. Muslim C. Protestant D. Catholic E. Others, specify________

5. Current marital status? A. Married B. Single C. Divorced D. windowed

6. Level of education?

A. BSc Nurse B. HO C. Lab Technologist D, Pharmacist (BSc)

E, Diploma Nurse F. Lab Technician G. Pharmacy Technician

H, BSc Midwifery I, Diploma Midwifery J, BSc Environmental health

K, Diploma Environmental health L, GP M, Others…………………………………

7. Year of experience………………

8. Currently working ward? ....

9. Do you have **Television** in your home? A, Yes B, No

10. Do you have **Radio** in your home? A, Yes B, No

11. Currently, are you a social media (facebook,……) user? A, Yes B, No

Part II: KNOWLEDGE ASSESSMENT (You can choose more than one answer)

12. Have you received any training/ orientation on COVID 19?

A= Yes B= No

13. Is COVID_19 at a pandemic or epidemic stage? A= Pandemic B= Epidemic

14. What is the call center service number to seek information about COVID-19 in Amhara region or Ethiopia?

A,-------------------- B, I don't know

15. What are the main clinical symptoms of COVID-19? **(Select all that applies)**

A, Fever B. Fatigue & myalgia C. Dry coughs

D. Shortness of breath E. Loss of appetite F. Common cold like symptoms

G. Others………………

16. Corona virus spreads via **(Select all that applies):**

A. Respiratory droplets of infected individuals during sneezing and coughing

B. Direct contact to infected person (hand shaking, skin to skin,)

C. Indirect contact to objects & utensils contaminated by corona virus (fomite, surface, etc)

D. Eating contaminated vegetables and uncooked meats

E. Air born F, Other………………………………….

17. Persons with COVID-2019 cannot transmit the virus to others when symptoms not present. A. Yes B. No C. I don't know

18. To protect ourselves from corona virus infection we should do**(Select all that applies)**

a. Frequent hand washing with soap
b. Maintaining at least 2 metre physical distance
c. Staying at home
d. Alcohol based hand rub (sanitizer's)
e. Avoiding visiting crowded places and mass gatherings
f. Following good respiratory hygiene
g. Isolation and treatment of the case
h. Avoid touching eyes, mouth and nostrils before washing hands
i. Using personal protective equipment's like face mask
j. Stay informed and follow healthcare workers advice
k. Others…………………………………………………………………

19. Using only one of the above methods is adequate to prevent corona virus infection (Example, N95 face mask only, OR only frequent hand washing)

A. Yes B. No C. I don't know

20. Which segment of the population is **at risk of getting** corona virus infection?

A, Geriatrics/Older peoples B, Children and young adults
C, All age group and both sex E, Men's
D, People with chronic diseases F, Women's
G, Other……………...

21. Which segment of the population appears to be more vulnerable to becoming **severely ill or die** with the virus? **(Select all that applies)**

A. All age groups and both sex
B. Older people/Geriatrics
C. Children and young adults
D. People with chronic diseases (such as asthma, diabetes, heart disease, obese)
E. Males only

F. Females only

G. Others……………………..

22. It is **not necessary** for children and young adults to take measures to prevent corona virus infection.   A, Yes   B. No   C. I don't know

23. When do you **suspect** that an individual might have a corona virus?

A, One of COVID_19 symptoms plus Travel history

B, One of COVID_19 symptoms Plus Contact History

C, One of COVID_19 symptoms Plus Occupational risk

D, COVID_19 symptoms only

24. How do we **confirm** that whether an individual is infected with a corona virus or not?

A, Through laboratory test   B, Using signs and symptoms

C, If he comes from abroad   D, Other………………………

25. If an individual is confirmed to have corona virus, then what activities the gov't has to do?

A. Isolation and treatment of the case
B. Contacts tracing and quarantining
C. Performing lab tests for contacts
D. Disinfection of houses and utensils of a COVID 19 case
E. Others…………………………………

26. A person who has contact with a confirmed COVID 19 case should be quarantined for how many days? ………………………………………..

27. Currently, is there any drug that can cure COVID 19 cases?

A, Yes   B. No   C. I don't know

28. Currently, is there a vaccine for the corona virus disease?

A, Yes   B. No   C. I don't know

29. All individuals infected with corona virus will die?

A, Yes   B. No   C. I don't know

30. Supportive treatments can help most patients to recover from COVID-19.

A, Yes   B. No   C. I don't know

31. Is there any person who died of corona virus in Ethiopia?

A. Yes   B. No   C. I don't know

	Attitude (Tick in the appropriate columns)	Agree	disagree	I don't know
1	Do you agree that corona virus will not infect or kill African origins or Ethiopians?			
2	Do you agree that COVID 19 only infects elders?			
	Do you believe that COVID_19 will not kill children and youths?			
4	Some people says that there is no need to bother about COVID 19 prevention strategies; because God/Allah will protect his creators from corona virus infection? Do you agree this idea?			
5	Do you think that avoiding mass gatherings (church/mosque, market, wedding, etc) will prevent corona virus infection?			
6	Do you believe that frequent hand washing and using sanitizers can prevent corona virus infection?			
7	Do you believe that staying at home can prevent corona virus infection?			
8	Do you believe that maintaining a social distancing can prevent corona virus infection?			
	Do you think that using N95 face mask only is adequate to prevent corona infection?			
9	Do you think that cultural medications like *Fetto, lemon, ginger, shinkurt, honey*, etc) can prevent and/or cure COVID 19 cases?			
10	Do you agree that COVID-19 is a preventable disease?			
	Do you agree that Ethiopia can win the battle against the COVID-19 virus?			
11	Do you advice corona virus prevention strategies for your friends and families?	Yes	No	
12	If your intimate friend recently comes from a COVID 19 affected countries without being quarantined for 14 days, can you report it to a police or heath office?	Yes	No	
13	If the price of corona virus prevention strategies (Soap, sanitizers, water etc) becomes too costly can you buy it?	Yes	No	

Part IV Practice part

1. Which preventive measures are you mostly practicing to protect yourself from COVID-19? [Tick all the option/s that is/are true for you]
    A. I avoided going to crowded places
    B. I avoided taking public transportations
    C. I avoided shaking hands
    D. I am keeping physical/social distance
    E. I avoided touching eyes, nose and mouth before I washed my hands
    F. I wash my hands frequently with soap and water or
    G. I am using alcohol-based hand rub
    H. I cover my cough using the bend of your elbow or a tissue
    I. I am staying at home
    J. Stay informed and follow advice given by your healthcare provider
    K. I use mask when leaving home or at my work place
    L. I am not practicing any preventive measures
1. In recent days, have you gone to any crowded place (church, mosque, market, etc)?
    A, Yes B. No
2. In recent days, how much times your hand washing behaviour has increased as compared to the era of no corona virus infection?
    A, Not increased B, two times C, Three times D, Four times
    E,……………….times
3. In recent days, how many times a day you wash your hands? ………………… times
4. Do you have a sanitizer in your pocket? A, Yes B, No
5. In recent days, how frequent are you using a soap for hand washing?

    A, Sometimes B, Always C. I am not using soap

9. Are you applying social distancing strategy to prevent corona virus infection?

    A, Sometimes B, Always C. No

10. In recent days, are you using a face mask in your working area?

    A, Sometimes B, Always C. No

11. In your health facility, is there a patient triage? A, Yes B, No

Name and signature of data collector _______________ ***Thanks for your cooperation***
